# Calibration of Displacement Laser Interferometer Systems for Industrial Metrology

**DOI:** 10.3390/s19194100

**Published:** 2019-09-22

**Authors:** Han Haitjema

**Affiliations:** Mechanical Engineering Department, KU Leuven, 3000 Leuven, Belgium; han.haitjema@kuleuven.be; Tel.: +32-1637-4283

**Keywords:** laser interferometer, calibration, traceability

## Abstract

Displacement laser interferometer systems are widely used for the calibration of machine tools and CMMs (Coordinate Measuring Machines). Additionally, they are often the workhorse in dimensional calibration laboratories, where they act as the basic metrological traceability link for many calibrations. This paper gives a review of the calibration of such systems, where several approaches, such as the calibrations of separate components or the system as a whole, are reviewed. The calibrations discussed are: the laser frequency, the counting system, software evaluation of the environmental conditions, environmental and material temperature sensor calibration and the calibration of optics that is part of the system. For these calibrations considerations are given about the ways these can be carried out and about establishing the re-calibration intervals.

## 1. Introduction

Displacement laser interferometer systems have entered the market since the early 1970s [1] and have become the de facto reference standard for dimensional measurements in which displacements are involved [2]. Together with this introduction and rapidly-spreading use the request for calibration, traceability and uncertainty of these systems raised automatically. The traceability matter was greatly advanced by the re-definition of the meter in 1983, especially where this was accompanied by documentation that described the iodine stabilized He-Ne laser, when operated under defined conditions, as a primary length standard [3]. Through this route, a comparison of the displacement interferometer laser light source to an iodine-stabilized He-Ne laser establishes a direct traceability to a primary standard and thus to the meter definition. Next to this frequency-stabilized light source, a laser interferometer displacement measuring system consists of a number of components that all may give deviations and contribute to the final uncertainty in various ways; therefore, it is generally considered that a frequency calibration of the light source on its own may be not sufficient to declare a system as ‘calibrated’ and/or ‘traceable’.

**Figure 1:**
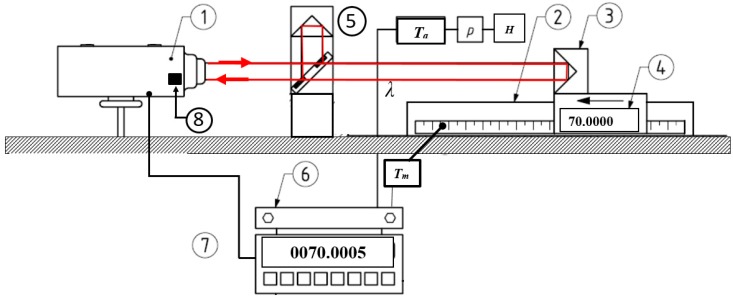
Example of a laser interferometer operation: 1: laser head, 2: slide way, 3: measurement corner-cube, 4: machine-axis reading, 5: beam splitter and reference corner-cube, 6: compensation unit, 7: displacement read-out and 8: photo-detector.

A displacement laser interferometer is based on the interference of a light beam that is emitted by a laser. Commonly, this laser is frequency stabilized. This stabilization can be based or two-mode or Zeeman-stabilization [4]. They can operate as homodyne or heterodyne interferometer, where, respectively, one and two frequencies are used. The operation principle and a comparison between these two principles were summarized by Cosijns [5] (see sections 10.2.1.1 and 10.2.1.2 in this reference). An example of a laser interferometer operation that illustrates its use and the features that might require calibration is given in Figure 1. The laser interferometer system uses the sensors as indicated in Figure 1 to calculate the displacement. Both homodyne (operation at a single laser frequency) and heterodyne (using two laser beams with a slightly different frequency) systems use in the end the following equation to calculate the measured displacement (see equation 10.15 in [5]):

(1) $$L=\frac{\left(N+\delta \right)\cdot \lambda_{v}}{2\cdot n_{a}\left(T_{a},p,H\right)},$$

where *L* is the displacement, *N* is the number of interference fringe-counts, *δ* is the fractional interpolation between counts, *λ_v_* is the laser wavelength in vacuum and *n_a_* is the refractive index of air that depends basically on the air temperature *T_a_*, the air pressure *p* and the humidity *H*.

When an object or a scale was measured, this measurement was corrected to 20 °C. This led to the basic equation for the operation of a displacement laser interferometer system:

(2) $$L_{i}=L\cdot \left(1-\alpha_{m}(T_{m}-20\;{}^{\circ}C\right))=\frac{\left(N+\delta \right)\cdot \lambda_{v}}{2\cdot n_{a}\left(T_{a},p,H\right)}\cdot \left(1-\alpha_{m}(T_{m}-20\;{}^{\circ}C\right)).$$

where *L_i_* is the indicated length, *T_m_* is the material temperature and *α_m_* the linear material expansion coefficient of the measured object or calibrated scale.

The calibration may consist of calibrating all individual variables in Equation (1), i.e., the vacuum wavelength (*λ_v_*), the counting system (*N*), the fractional interpolation (*f*), and the sensors that measure the air conditions (air temperature *T_a_*, pressure *p* and humidity *H*), as well as the material temperature *T_m_*.

However, such a calibration of all the components does not ascertain that these were evaluated properly by the compensation unit and the displacement read-out. For the vacuum-wavelength, which corresponds to the laser frequency ν by $\lambda_{v}=c/\upsilon$, where c is the velocity of light in vacuum, it is essential that the nominal vacuum wavelength, i.e., the vacuum wavelength that the system uses in its calculation, is known in order to conclude if there is any deviation or not. A calibration of the optics used, i.e., the corner cubes and the beam splitter, is possible and can be useful when (sub-) nm accuracies are needed. However, these are not commonly carried out. Still, a calibration will be useful for special optics for additional measurements, such as rotations or straightness.

A calibration, or test, that the system operates well as a whole is often performed in addition to the calibration of the separate components. One can even argue that the calibration of the system, e.g., against a calibrated laser interferometer system, may replace the calibration of at least some of the components that determine the final measurement uncertainty. 

Calibrations are commonly performed in national metrology laboratories (NMIs), but there is no principal limit or prohibition that (accredited) calibration laboratories perform at least some of the calibrations and tests that ascertain the reliability of laser interferometer systems. Users can perform some of the tests as well, for example, as intermediate check between calibrations. In the remainder of this paper, calibration methods for the components as well as the system are described and discussed. This paper reviews the calibration of commercially available systems that have been designed for general use in industrial and calibration laboratories. These are commonly referred to as “laser interferometer”, “calibrator”, “laser calibration system”, “optical laser encoder”, “laser interferometry system”, “displacement measuring interferometer systems” or “laser transducer system”. The common aspect is that displacements are measured by interferometry. 

## 2. Laser Frequency Calibration: *λ_v_*

### 2.1. Calibration Against an Iodine Stabilized Laser

**Figure 2:**
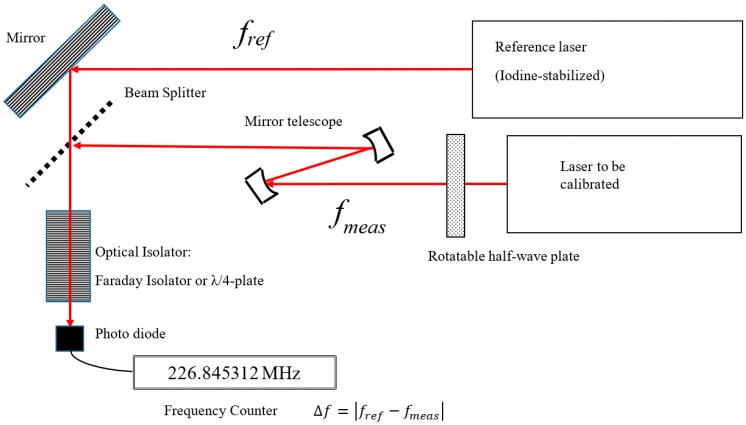
Scheme of a typical set-up for laser frequency calibration.

The laser vacuum wavelength is commonly calibrated by a so-called beat-measurement: the laser beam is mixed with a laser beam of an iodine-stabilized He-Ne-laser that acts as a primary length standard. This is a major, and in many cases the only, application of an iodine-stabilized He-Ne laser that is present as the primary realization of the meter in many national laboratories. The typical set-up is sketched in Figure 2 [6,7]. The laser beam to be calibrated is mixed with the laser beam of the reference laser. When the beams are well aligned, interference between these beams occurs with a frequency that is equal to the frequency difference between the beams. The half-wave plate is used to align the polarization direction of the laser to be calibrated to polarization direction of the reference beam, so that interference is effective. The telescope mirrors are intended to reduce the beam diameter to be equal to that of the reference laser, so that the interference intensity is optimal. The optical isolator is intended to prevent optical feed-back to the laser; especially iodine-stabilized lasers can be very sensitive to this. 

Iodine-stabilized lasers have some positive and negative properties that affect the comparison to be more or less convenient. Already mentioned are the relatively low power (typically 75–125 μW) and the high sensitivity to optical feedback. Another characteristic is that the frequency is modulated with an amplitude of 6 MHz, at a frequency of typically 5 kHz, which makes averaging over some time (typically 10 s) necessary in order to get stable results, and the frequency difference must be significantly larger to obtain useful results. On the other hand, a very useful feature is that the iodine-stabilized laser can be stabilized at several different hyperfine transitions of the iodine molecule. The corresponding frequencies are labeled as *a* through *t* for the commonly used isotope and transitions and are defined in the meter definition documents [3,8]. By stabilizing the reference laser on different transitions, the sign of the frequency difference can be established. Without this feature, it would be hard to discriminate between a higher or lower frequency of the one laser relative to the other, although considering the sign of the modulation coefficient of the lasers, it could be an option.

The iodine-stabilized laser is mentioned as a primary standard in the earliest documents concerning the meter definition based on the speed of light from the BIPM (Bureau International des Poids et Mésures) [3]. Since then, several comparisons have been held (e.g., [9]) that led to the conclusion that the quoted uncertainty of this standard could be further reduced, as was established in [8,10]; this was reconfirmed more recently [11]. This is summarized in Table 1.

**Figure 3:**
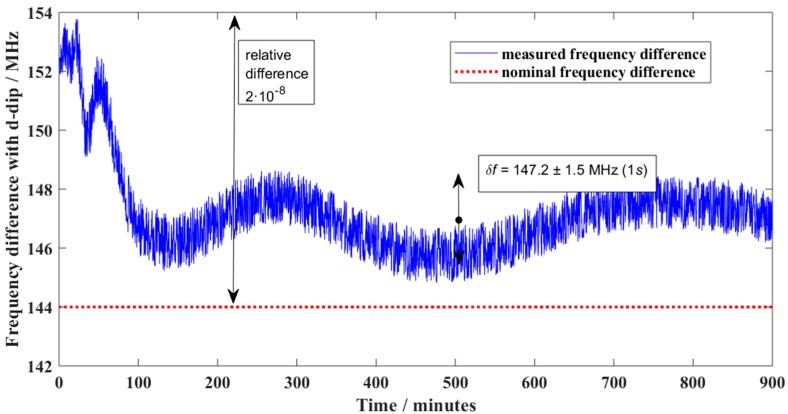
Typical result of a beat-measurement between an iodine stabilized laser and a laser used in a displacement laser interferometer system. The uncertainty of the iodine stabilized reference laser is better than 0.05 Mhz; this corresponds to a thin horizontal line on this scale; its stability is even better.

Table 1 shows that the relative uncertainty of the iodine-stabilized laser is now smaller than 1 × 10^−10^. This is negligible compared to commonly required uncertainties (1 × 10^−7^ to 1 × 10^−8^) of laser interferometer systems. The calibration commonly consists of recording the beat frequency during some time, typically a few hours, after the laser to be calibrated indicates that it is stable. This can be repeated a few times—typically 3 times or more—to see if the frequency is consistently within the same range. The result of a typical measurement is shown in Figure 3. The dataset presented in this figure can be foud in Supplementary Materials.

It can be discussed how many measurements should be taken, how long these should last, how many ‘cold re-starts’ of the laser should be performed etc. Some considerations were given by Lipus [12] who concluded, based on considerations of noise, repeatability and the Allan-variance, that 10,000 samples of 10-second long period (28 h) is an appropriate value. In addition, three different ‘cold starts’ followed by one hour measurement time can be taken to determine the range of maximum deviation that commonly appears in the first hour after start-up. Normally, an accredited laboratory will have a standardized procedure, based on the considerations above. It should be kept in mind that under normal laboratory conditions in air, the frequency and its stability is the least significant contribution to the measurement uncertainty of a laser interferometer system when compared to uncertainties in the measurement of environmental conditions, and due to alignment. Some evaluation possibilities are:

A. The mean frequency and the standard deviation are evaluated over longer times and multiple start-ups. The mean frequency (or wavelength) that is achieved after some hours of operation is given as a systematic deviation of the system and the standard deviation from this value is given as the standard uncertainty that should be taken into account when the system corrects for this systematic deviation. For systems that enable to input or change the nominal vacuum wavelength it uses, this is a viable option. In terms of measurement uncertainty, this is a type A approach. To this, the uncertainty of the reference (*u* = 50 kHz or less) should be added quadratically; however, this value is commonly negligible compared to the observed deviations. 

For the measurement depicted in Figure 3 this would imply the following calculation:

$$\begin{array}{cc} \mathrm{The}\;\mathrm{absolute}\;\mathrm{frequency}\;f_{meas} & =473,612,379.8\;\mathrm{MHz}\;\left(d-\mathrm{dip}\right)-147.2\pm 1.5\;\mathrm{MHz}\;\left(\mathrm{measured}\;\mathrm{difference}\right)\\ & =473,612,232.6\pm 1.5\;\mathrm{MHz} \end{array}$$

where the uncertainty is given as a single standard deviation. From the manufacturers specification, it is known that the nominal vacuum frequency is 473,612,236 MHz, thus, the deviation can be expressed as ∆*f* = −3.4 ± 1.5 MHz. It is most appropriate to record the relative deviations, as the deviations affect the length measurements in a proportional way as well.

B. The maximum deviation from the nominal frequency over all the measurements is taken as the basis of the uncertainty, where no correction to the length measurement is made. From Figure 3, it can be seen that the maximum relative deviation from the nominal frequency is 2 × 10^−8^, giving a standard uncertainty of *u* = 2 × 10^−8^/√3 = 1.2 × 10^−8^. In terms of measurement uncertainty, this is a type B approach, where the repeated measurements are used to determine a maximum observed difference from the nominal value. Additionally, here, the uncertainty of the reference (*u* = 50 kHz or less) should be added quadratically, which will commonly be negligible compared to the observed deviations. 

In this case, the result can be reported as in Table 2. For heterodyne interferometers, the laser beam contains two frequencies that are mutual orthogonally polarized and have a fixed and stable difference of around 3 MHz for Zeeman-stabilized systems and may vary to values up to 10 MHz for systems that use AOMs (Acousto-Optic Modulators). With the set-up as sketched in Figure 2, these can be measured separately by rotating the half-wave plate such that either of them is calibrated. One can either do two separate frequency calibrations, or quickly switch between them, or measure the difference once and correct the calibration value as to obtain the mean frequency, or just measure one frequency and incorporate the other frequency in the uncertainty.

Depending on the orientation of the reference, e.g., the reference corner cube (5) in Figure 1, either frequency can be used in a measurement and it is usually not feasible to distinguish between the beams. As 3 MHz corresponds to a relative difference of about 6 × 10^−9^ in a length measurement, this is commonly not significant. 

More elaborate considerations are given by Lee [13], who concluded from a large amount of laser interferometers calibrated over some decades that, in general, the relative wavelength deviation is within 1 × 10^−7^ from the nominal value, the stability remains well within the 10^−8^ level, and the drift over years may be a few times 10^−8^ but not much more.

As practical information to the reader, Table 3 gives the nominal wavelength of several laser interferometer systems. 

An even more accurate reference frequency can be obtained using a frequency comb that gives a direct connection to the realization of the second and takes down the uncertainty from the 10^−11^ to the 10^−12^ level [14]. Although an iodine-stabilized laser is less suitable for using in a displacement laser interferometer directly, it is possible to establish a direct link to a primary standard when operating a laser interferometer [15]. In that case, a calibration as mentioned in this section becomes obsolete. 

### 2.2. Calibration Against Another Stabilized Laser

The iodine-stabilized reference laser as depicted in Figure 2 can be considered as quite some overkill considering its limited contribution to the uncertainty (typically 50 kHz or less) when compared to the laser stability (up to some 10 MHz, see Figure 3) and other uncertainty sources (see next sections). Therefore, it can be argued that another laser, calibrated and stable at the 10^−8^ level, could be used as well, inserting another step in the calibration chain. When a heterodyne laser is used [16,17], the frequency difference between the beams—if well recorded and documented—can be used to establish the sign of the frequency difference. 

### 2.3. Omitting Calibration by Considering the Laser as a Primary Standard

As an He-Ne laser operates by physical principles, the wavelength and frequency range it can possibly emit is limited. In 2009, it was recognized that the definition of a primary standard based on this fact can be useful, even with a relative low uncertainty [18]. This means that a frequency *f* = 473.6127 THz, corresponding to a vacuum wavelength *λ_v_* = 632.9908 nm, can be attributed with a relative standard uncertainty of 1.5 × 10^−6^ to any free-running or stabilized ‘red’ He-Ne laser. This means that one can refrain from calibration if this uncertainty is sufficient for the application. For example, for displacements within a 30 mm range, this gives a standard uncertainty of 0.045 μm that will be sufficient for, e.g., dimensional measurements of small plugs and rings and even gauge blocks.

The relative uncertainty of 10^−6^ was already mentioned in a letter from the NBS (National Bureau of Standards) on laser interferometer systems dated October 18, 1976, which stated that “modern stabilization techniques (...) cannot by misfunction degrade the performance below the 10^−6^ level”. This letter was reprinted in [19].

### 2.4. Indirect Calibration by Comparing to a Reference System

By generating a displacement that is simultaneously measured by a reference laser interferometer system and a system under test, the tested system is calibrated directly, and its wavelength indirectly. This is part of the calibration on a test bench that is further treated in Section 3.

## 3. Counting System: The Comparison Test Bench: *N*

A laser interferometer system can measure large displacements by accumulating (counting) light-dark transitions: *N* in Equation (1). If the intensity drops because of misalignment or a diverging laser beam, the system must give a warning and stop counting. Additionally, the hard-and software processing must not make mistakes in a proper counting, as a user may not notice it when some counts at λ/2 ≈ 0.3 μm or less (e.g., λ/8) are missed or lost. For very large displacements (>10 m), the counting system may overflow or suddenly start counting backwards.

The appropriate method for testing this is using a test bench where a same displacement is measured by both a reference laser interferometer system and the system under test. For such a set-up many variations have been proposed, depending on the measurement principle (e.g., one system uses a non-polarizing beam splitter optics, the other uses a polarizing beam splitter) or the basic philosophy that a system must be calibrated including its own optics (more on that later). In national metrology institutes, the test bench that was already present for measuring long tape measures is often used or adapted for comparing laser interferometer systems.

**Figure 4:**
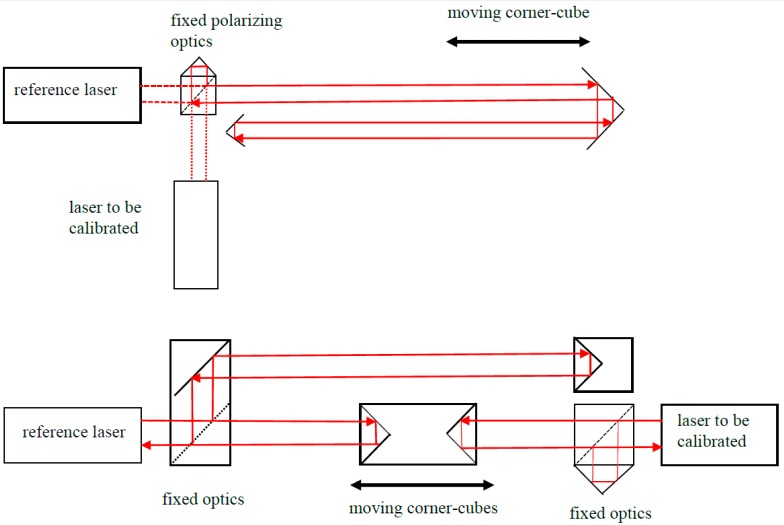
Two configurations for a comparison configuration. Top: with folded beam and joint light path and optics; bottom: compensated back-to-back comparison.

Already in 1982, Schellekens proposed a set-up that enables each system to use its own optics, while measuring the displacement simultaneously [6]. Schüssler [20] extended this to a two-sided system and he even proposed a system for calibrating four systems simultaneously. The range can be extended by folding beams inside a set-up that was proposed by Schüssler [20] and also used by Sparrer [21] and Wedde [22]. Stone [23] improved the two-sided system by an automatic compensation for the potentially different air conditions in the two branches, which was also proposed in ASME B89.1.8-2011 [24] (ASME is the American Society of Mechanical Engineers). Figure 4 gives two typical set-ups: a folded beam where the systems use common optics and a compensated back-to-back system. The part with common optics is preferred as long as the beams do not affect each other, i.e., the polarizing beam splitter must separate both polarization directions well. If any problems because of mixing occur, one of the systems may be rotated 90 degrees, or may be configured to use one central path for both the emitted and returning beam.

The moving part must be moving reasonable straight, preferable within 1 mm straightness deviation in order not to lose the signal while moving, and over a length where the system has to be calibrated; from a few m to 50 m in extreme cases. The alignment of both systems is critical as the comparison is critical to cosine errors; for example, a 1 mm vertical deviation over *l* = 4 m displacement gives a relative cosine error of 3 × 10^−8^ × *l*. The back-to-back comparison has an additional issue when the moving corner-cubes assembly rotates. This was analyzed in detail by Tang [25]. 

The comparison system can be used for a multitude of calibrations and test, just for this section we concentrated on the counting system. For testing the counting system, both systems should be used with the environmental compensation disabled, as if they were measured in vacuum. If this is not directly possible in the software this may be achieved by putting first the humidity to zero, and then the air pressure. Additionally, the material temperature correction must be disabled by defining the material temperature as *T_m_* = 20 °C.

The counting system was tested by moving the corner cubes slowly from the minimum to the maximum reading—and back—and recording the differences. It is common to zero both systems at a minimum position and to record some additional 10 measurement positions within the measurement range. This moving can be automated well [26]. The readings must be consistent with the frequency calibration of both the reference and the tested laser. Remaining influences are noise, cosine errors and interpolation errors. If the reading differences do not exceed these expected influences the counting system can be regarded as ‘OK’, i.e., the system is able to count properly up to the maximum displacement that is generated. Counting failures may result in sudden jumps in the values of the order of λ/2 to λ/8, and not coming back to the same value when returning to the original position. If the nominal frequency of the tested laser is unknown, the deviation from the nominal frequency can be estimated from the deviation from the reference laser that is proportional to the displacement. Together with the calibrated frequency according to Section 2, the nominal frequency of this system may be reconstructed. The frequency stability of the tested laser may be tested indirectly by keeping the system at its maximum displacement (the farthest position of the measuring corner cube) and noting the fluctuations in the measured length differences, assuming that the reference laser is more stable.

To illustrate a nominal-wavelength reconstruction, the frequency calibration given in Section 2.1 is taken, combined with a length difference measured by this laser on the test bench. 

The reference laser interferometer indicates 6910.68256 mm displacement; the laser under test indicates 6910.68233 ± 0.00007 mm. The frequency of the laser under test is 473612232.6 ± 1.5 MHz; that corresponds to a vacuum wavelength of λ_v_ = 632.9913743 ± 0.000002 nm. 

The laser under test measures 0.00023 mm too short over 6.9 m displacement; this means that the nominal vacuum wavelength is relatively 0.00023/6910.68256 smaller than the actual wavelength λ_v_ = 632.9913743; thus, the nominal wavelength must be:

λ_v,n_ = 632.9913743 × 6910.68233/6910.68256 = 632.9913532 ± 0.000006 nm

Here, the uncertainty is mainly limited by the test bench (noise and cosine-errors).

If two heterodyne systems are available that have a similar frequency difference between the reference and measurement beam, there is another method that may show the ability of the systems to count large displacements. As the frequency differences of two systems are not exactly the same, a system may start counting when receiving the beam containing the two frequencies from the other system. This means that this system in fact measures a velocity. It can be checked whether this velocity is detected as constant, and the counting system can be observed to check if it measures a continuously increasing or decreasing value, and its maximal detectable displacement may be observed. For example, one system stopped counting at 10.6 m. Another system counted up to 40 m, and then continued to count from −40 m to zero. Whether a system can really measure such displacement depends, next to the software counting capability, on the beam divergence and the corresponding decreasing signal strength.

**Figure 5:**
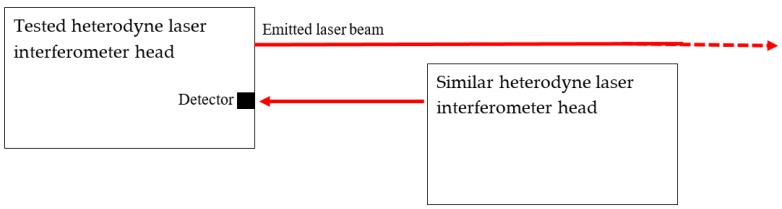
Checking the counting system of a heterodyne laser interferometer using a similar laser interferometer to generate a detected speed.

Figure 5 depicts the—rather straightforward—set-up for this test.

## 4. Short Range Interpolation and Nonlinearity Errors: δ

**Figure 6:**
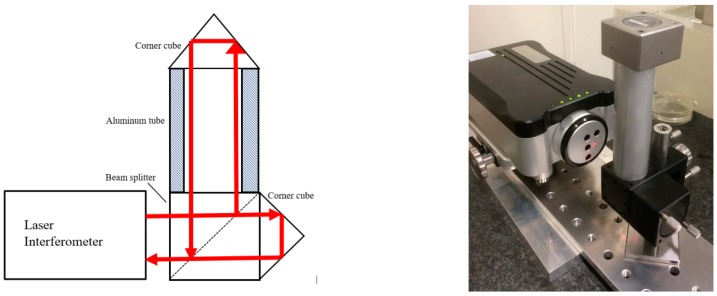
Schematic and picture of a set-up to determine interpolation errors by letting an aluminum tube cool down.

It is known that laser interferometer systems exhibit linearity errors due to the interpolation between fringes, the δ in Equation (1). These have been intensively studied and modeled in literature [27,28,29]. In these studies on periodic linearity, usually a third-order polynomial or several harmonics of a Fourier transform are used to describe the deviations. In general, it can be concluded that under normal circumstances (well aligned system, optics as supplied with the system) these deviations are of nm-level. Normally the set-ups made for calibrating large displacements as described in Section 3 are not accurate enough to detect such deviations. The calibration of nm-level displacements is a subject on its own, where the techniques may vary from X-Ray interferometers [30] and Fabry-Perot interferometers [31,32] to separated beams in a heterodyne interferometer [33]. In the case of laser interferometers the knowledge that the non-linearity must be periodic with a period of λ/2 can be used to simplify the calibration. For example, the displacement can be calibrated against a PZT (Lead (Pb) Zirconate Titanate) device in a small range, where the typical periodic deviations from the laser interferometer can be separated from the PZT-non-linearity that will have a more monotonic behavior [34], or it can be calibrated against a reference laser for which the periodic deviations are already calibrated. When an uncertainty of a few nm is sufficient and more specialized equipment is not available an effective simplified calibration can be carried out by using a slightly warmed-up aluminum tube and assuming that the length changes in a monotonous way while the tube cools down to the environmental temperature [35,36]. Because of the high linear expansion coefficient and the high thermal conductivity of aluminum the tube exhibits a significant length change, while the air inside the tube shows little turbulence. The length change as a function of time can be fitted by a polynomial, and the deviations from linearity can be identified as non-linearities caused by the laser interferometer system combined with its optics. As the method introduces some noise as well, it determines an upper limit, which may be sufficient for laser interferometer systems considered here. Figure 6 gives a picture and a schematic of this set-up. 

**Figure 7:**
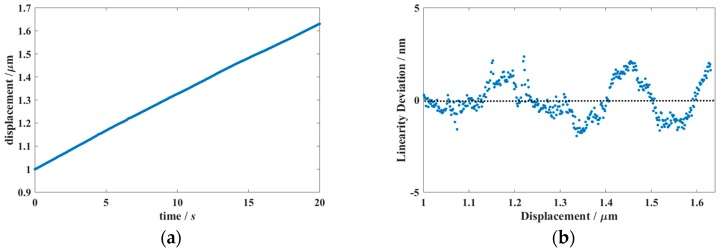
(**a**) Displacement as a function of time during cooling down of the aluminum tube. (**b**) The linearity deviation from a second-order fit.

A displacement is generated by warming up the tube by hand and letting it cool down to room temperature. During the cooling-down period the displacement is recorded with a sampling frequency of 20 Hz as a function of time; see Figure 7a. A second-order fit is made to the time as a function of the displacement. The deviations from this fit were recalculated to displacements and these were plotted as linearity deviations as a function of the displacement, see Figure 7b. The figure shows that for this example the linearity deviations are within ±2.5 nm. This dataset can be found in Supplementary Materials. The literature reports even smaller values for linear displacement systems based on corner cubes [34].

Noise and drift of the system can be determined by redirecting the measurement beam directly into the detector by one single corner cube only; without any beam splitting optics. Although some drift can be observed in older systems, modern systems show hardly any noise and no drift beyond the resolution of 1 or 10 nm. This can be an efficient alternative to the determination of the zero-drift as described in Section 4.6.1 of ASME B89.1.8-2011 [24], where deviations in the calibration set-up may have more effect than the zero-drift of the system itself.

When upper limits of the noise and nonlinearity are determined with tests as described in this section, further calibrations using the comparison test bench as described in Section 3 can concentrate on effects proportional to the length such as temperature, refractive index, etc., and the use of ‘own optics’ is not needed as this is already covered in the short-range test. Without the non-linearity test, considering the literature about non-linearity errors, it is safe to take a value of ±10 nm as maximum linearity deviation, giving a standard uncertainty of *u* = 10 nm/√3 = 5.7 nm.

## 5. Software Checks on Environmental Conditions: *n_a_,T_m_*

A laser interferometer system uses software to correct for the environmental conditions. 

Concerning the air temperature, pressure and humidity, it must be checked whether the refractive index ‘Edlen’ equation is used properly by the system software. It is generally agreed that the equations given by Birch and Downs [37], with a correction for the CO_2_ according to Muijlwijk [38], Bönsch [39] and Ciddor [40], give the most appropriate values, with an inherent relative standard uncertainty of about 2 × 10^−8^. Most convenient is the NIST (National Institute of Standards and Technology) reference software that is available online [41]. For reference conditions *λ* = 633 nm, *p* = 1013.25 hPa, *T_a_* = 20 °C, CO_2_-content = 450 ppm, the results are:
Birch and Downs:  *n_a_* = 1.000271376Ciddor  *n_a_* = 1.000271373Bönsch  *n_a_* = 1.000271374

The correspondence is within 3 × 10^−9^, the standard uncertainty put to the equations is about 1 × 10^−8^. This is neglecting the sensors deviations and uncertainties, these are treated separately. Sensitivities are: for air temperature: *dn_a_/dT_a_* = −9.6 × 10^−7^/°C; for air pressure: *dn_a_/dp* = 2.7 × 10^−7^/hPa; for humidity *dn_a_/dH* = −8 × 10^−10^/Pa = −8 × 10^−9^/%Rh; for CO_2_-concent: *dn_a_/dx* = 1.5 × 10^−10^/ppm.

There can be quite some difference between manufacturers and software versions, thus, the software needs to be checked in one of the ways described below; usually, the way described in Section 5.1 is sufficient.

### 5.1. Indirect Software Check

The refractive index as it is calculated with reference software, for a set of given input data, is compared to the refractive index as it is derived from the laser interferometer software data. The latter may be the HP (Hewlett-Packard)/Agilent VOL (Velocity-Of-Light)-factor, (*n*^−1^ = 0.999 + VOL × 10^−6^) or a correction factor *c*_a_: *n_a_* = 1/*c_a_*, or directly, *n_a_*. Other systems give the wavelength or half a wavelength in air: *λ_a_ = λ_v_/n_a_*.

The resolution of the indicated value of the wavelength and/or *n* may be limited to 1·10^−7^; in that case, the resolution may be increased by checking by what change in, e.g., pressure or temperature the last digit flips. ASME B89.1.8.2011 [24] recommends that nine combinations of mean and minimum/maximum values of air temperature, air pressure and humidity are checked. 

For example: the software indicates a wavelength *λ_v_* = 632.99058 for vacuum conditions. For the reference conditions as stated above the software indicates *λ_a_* = 632.81890. This gives effectively for the *n_a_* calculated by the software: *n_a_* = *λ_v_/λ_a_* = 632.99058/632.81890 = 1.00027129. Compared to the reference value 1.00027137, the deviation in *n_a_* is ∆*n_a_* = −0.00000008 ± 0.00000002.

Table 4 shows how the result and its effect on the measured length can be reported. If the deviation depends on the used parameters, this must be taken into account in the uncertainty.

### 5.2. Direct Software Check

A direct check on the software processing of environmental data can be made with manual input of the environmental data, using the comparison set-up as displayed in Figure 4. The reference laser interferometer remains in the ‘vacuum’-measurement condition and is used as monitoring device for the drift. For the laser to be calibrated, first, it is configured for vacuum conditions. Using the common light path comparison set-up, both systems are zeroed with the carriage at the maximum position; then the carriage is returned as close as possible to zero. Now, both systems display a large displacement, which is relatively stable, as the path through air is minimized. In this position, various environmental conditions can be input to the laser under test, and the different readings are recorded. These can be corrected for the environmental drift as it is monitored by the reference laser interferometer. An example is given in Table 5.

From the data in Table 5, the effective refractive index is calculated as:

*n_a_* = 6988.25646/(6986.36065 + 0.00048) = 1.000271290

This result can be reported as in Table 4.

### 5.3. Indirect and Direct Check of Material Temperature Compensation

The length *L* should be corrected to 20 °C material temperature according to Equation (2) that is repeated here:

$$L_{20}=L\cdot \left(1-\alpha_{m}(T_{m}-20\;{}^{\circ}C\right))$$

where *L* is the measured displacement without material temperature compensation, *T_m_* is the material temperature and *α_m_* is the linear expansion coefficient of the measured material.

This can be checked similarly as in Section 5.1 or Section 5.2 by varying *T_m_* and/or *α* within realistic ranges. A single check on this suffices and needs hardly to be reported, as only rather simple multiplications are involved and, contrary to the refractive index, there is no any debate or history on what equation is correct.

### 5.4. Complete System Check

A complete system check can be made by attaching the sensors to the laser system and carrying out the check as described in Section 5.2. Instead of manual inputting the environmental factors, these are now taken from sensors. This makes this evaluation less flexible for changing factors one-by one, but it best represents how the system is used in practice.

## 6. Environmental and Material Temperature Sensor Calibration: *T_a_*, *T_m_*, *p*, *H*

Environmental and material temperature sensor calibrations are essential elements of a laser interferometer system calibration. These calibrations as such are not considered to be part of the dimensional metrology expertise, and therefore, it may happen that the laser interferometer systems are taken to the temperature-, pressure- and humidity sections. As some systems do not operate with a laser attached, or might not even have a laser signal at all, this may give practical issues. If the issued certificates are just added to the frequency- and other calibrations, this may be cumbersome for the user who has to evaluate the meaning of these calibrations for the system performance.

Another approach can be that the sensors (air temperature, material temperature, air pressure and humidity) are calibrated using reference sensors in the conditions where, e.g., the comparison on the test bench was carried out, i.e., calibration on ‘one point’. This may be justified when the system will be used in a dimensional metrology laboratory anyhow, with nominal temperatures around 20 °C and humidity around 50%. The next section will discuss some of the typical characteristics of these sensors and their calibration

### 6.1. Material Temperature Calibration: T_m_

Material temperature sensors are typically shielded from the environment and may contain a magnet as to attach to typical steel measures (a gauge block, a scale or a work piece), as these are measured in dimensional metrology laboratories. It is logical to treat these as standard temperature sensors, i.e., calibrate them in a thermostatic bath at least three points in a requested range, which may be between 10 °C and 30 °C for sensors to be used in the field for the calibration of a variety of machine tools. Purists may argue that putting them ‘under water’ may affect the reading and does not represent the conditions under with these sensors are used later. Sometimes, the system enables adjustment/correction of the recorded temperatures; in that case, it is good practice, and a requirement for traceability, that the calibration values before and after adjustment are given.

**Figure 8:**
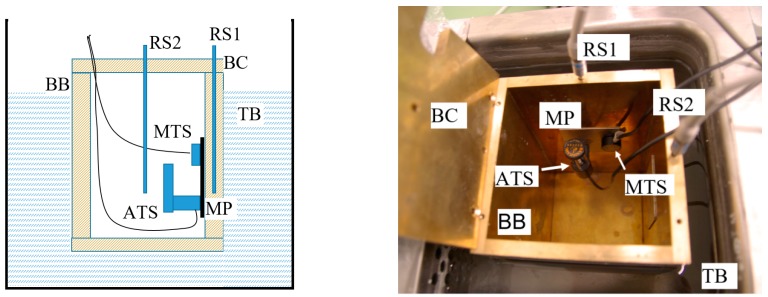
Schematic and realization of a calibration set-up for temperature sensors. TB: thermostatic bath; BB: brass box; BC: brass cover; MP: metal plate; RS1,2: reference Pt100-sensors 1; MTS: material temperature sensor; ATS: air temperature sensor.

A calibration set-up in which the sensors are calibrated as much as possible ‘like they are used’ is sketched in Figure 8.

The set-up as sketched in Figure 8 can be used to calibrate temperature sensors between 10 °C and 30 °C with an uncertainty of about 0.01 °C.

The thermal expansion coefficient *α_m_* must be known or estimated by the user.

For steel with *α_m_* = 11.5 × 10^−6^/°C, a temperature uncertainty of 0.02 °C corresponds to 2 × 10^−7^ × *l*. Such an effect on the length measurement uncertainty is a factor of 10 worse than a typical laser frequency stability or deviation, and also a factor of 10 worse than the counting system calibration. Table 6 illustrates how a calibration result can be usefully reported.

### 6.2. Air Temperature Calibration T_a_

The measurement of air temperature and the calibration of air temperature sensors are not trivial. The sensor may be affected by self-heat, especially in still air, while highly turbulent air may cool down the sensor. Additionally, it may be affected by radiation from distant heat sources. For the calibration the set-up of Figure 8 may be used. One rather infamous air temperature sensor, at least when it comes to calibration, is the Agilent/HP 10751A air sensor. This system heats up the air in order to generate an air flow. The sensor is mounted were the air comes into the sensor body, but even there the air is already heated up 0.8 °C so that a correction must be made which is already incorporated in the software. This correction vanishes when this system is calibrated in a climate chamber with a high air flow itself. On the other hand the calibration in a set-up like in Figure 8 fails because the whole interior is heated up by the sensor. A practical compromise is to calibrate this sensor in the laboratory environment using a reference sensor close to the sensor to be calibrated. This may give a correction value for calibrations taken else way, e.g., in a climate chamber. Table 7 illustrates how a calibration result can be usefully reported.

### 6.3. Air Pressure Calibration: p

The indication of the pressure indicator in the laser interferometer system can be compared to a calibrated reference sensor in the same room at the same height from the floor (vertical gradient is 0.1 hPa/m). This can be repeated several times over a day in order to check the repeatability of the system. For a broader range this calibration can be carried out in chamber that has a pressure controller; however, the cables from the laser interferometer system may be difficult to feed through. If time is not an issue, one can keep the system in one room for some three weeks; in that time, usually the weather has changed enough for a decent pressure range. Because of the costs and difficulties of a separate pressure chamber, it is rather common to carry out the calibration just at the air pressure in the laboratory at the time of calibration. Table 8 shows a typical calibration result and the effect on a length measurement. The effect on the length measurement is rather limited considering the accuracies by which pressure sensors can be calibrated. On the other hand the pressure indicator is the most risky element of a laser interferometer system; major temperature deviations are easily noticed as the users can sense these themselves, but major pressure deviations, e.g., due to a defective sensor after the sensor was dropped, are not automatically noticed by common sense. A quick intermediate check can be made by considering the reported atmospheric pressure in a weather overview, or obtain it from a near airport.

### 6.4. Air Humidity Calibration: H

In principle a humidity sensor can be calibrated in a climate chamber like a pressure- or temperature sensor. It must be kept in mind that the sensitivity of the length indication to the humidity is rather low. Some laser interferometer systems even just require that the user estimates the humidity to be ‘low’, ‘average’ or ‘high’, which corresponds to 25 %Rh, 50 %Rh and 75 %Rh, respectively. It can be argued that for the humidity a calibration at a single value in the laboratory may be sufficient. Table 9 illustrates how a calibration result can be usefully reported. 

## 7. Calibration of Laser Interferometer Optics

Part of laser interferometer systems is the optics. The standard operation mode is using linear optics; however, angular and straightness optics may also be supplied and require calibration. The principles and use of these optics was first described in 1983 [42] and has not principally changed since then. This section describes some tests and calibrations that may be performed using this optics.

### 7.1. Calibration of Linear Optics

The calibration of linear optics in combination with the system was already described in Section 4. A linear optics set usually consists of two retroreflectors (corner-cubes) and a (polarizing) beam splitter. Users often use a laser interferometer combined with several linear optics sets, some of which may be fixed in measurement instrument or calibration set-ups. Even optics of the one manufacturer may be used for a system of another manufacturer. Therefore, the value of calibrating the system ‘including own optics’ may be limited. What can be calibrated/checked about retroreflectors is whether their reflection angle deviates from 180°; this can be done using an autocollimator or a Fizeau interferometer. The polarizing properties of the beam splitter may be measured as well; this is of limited use, as the effect must be combined with the properties of the laser output, and even then, the estimation of the effect on a length measurement is far from straightforward. When the configuration contains a flat mirror, the flatness of this mirror may be calibrated using a Fizeau interferometer.

### 7.2. Calibration of Angular Optics

**Figure 9:**
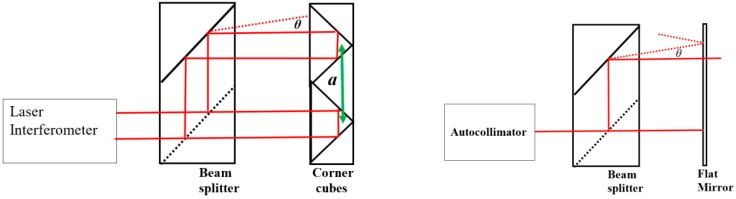
Left: angular optics assembly. *a*: characteristic distance between central-symmetric points *θ:* angular deviation of beam splitter assembly. Right: calibration of the angular deviation *θ*.

Angular optics are mainly used for measuring rotary deviations of guideways, but can also be used as rotation-measuring device for angles up to ±15°. This operation and the calibration method for a potential alignment error are depicted in Figure 9.

Figure 9 (left) gives the typical configuration of the angular optics; the figure at the right gives the configuration for the calibration of an important characteristic, especially when the beam splitter/mirror configuration is assembled separately. This deviation will appear as an angular difference between the beams transmitted and reflected in the beam splitter. Instead of the autocollimator, a flatness interferometer, typically of the Fizeau type, may be used as well.

The rotation angle *φ* of the corner cubes assembly when a displacement *d* is measured is given by:

(3) $$\sin\varphi =\frac{d}{a}\;\mathrm{so}\;\varphi =\arcsin\left(\frac{d}{a}\right)$$

The angular deviation *θ* gives a cosine deviation when the corner-cubes assembly is displaced over a length *l* without any rotation. The upper beam measures a larger displacement than the lower beam which is translated in an apparent angle *φ_a_*:

(4) $$\varphi_{a}=\frac{1}{2}\theta^{2}\cdot \frac{l}{a}$$

This deviation is linearly proportional with the displacement *l* and quadratic with the parallelism deviation *θ*. A parallelism deviation of 10″ gives a deviation of 3.5·10^−8^ rad = 0.008″ for *a* = 32.5 mm and *l* = 1 m; thus, in most cases, this will be negligible.

The angle calculation and calibration in Equation (3) gives little problems for small angles ($\varphi \approx \frac{d}{a})$. A calibration can, e.g., be carried out using a (long) sine bar, a technique that has been well developed for uncertainties in the 0.01″ region [43]. For larger angles, various methods have been developed, e.g., based on calibrated rotary tables [44]. Still, when carrying out a calibration for larger angles, the angle between beam splitter- and the corner cube assembly at the moment the system is zeroed, *φ*_0_ becomes relevant. In that case, it can be written that:

(5) $$\varphi =\arcsin\left(\frac{d+a\cdot \sin\varphi_{0}}{a}\right)-\varphi_{0}.$$

The characteristic distance *a* can be found by rotating the assembly from the zero position to two rather large angles *φ*_1_ and *φ*_2_, using a calibrated rotary table and recording the measured distances *d_1_* and *d*_2_. As Equation (5) is valid for both measurements, *φ*_0_ can be eliminated using:

(6) $$\varphi_{0}=\arctan\left(\frac{d_{2}\sin\varphi_{2}-d_{1}\sin\varphi_{1}}{d_{1}\cos\varphi_{2}-d_{1}-d_{2}\cos\varphi_{1}+d_{2}}\right),$$

and the characteristic distance can be found from:

(7) $$a=\frac{d_{1}}{\sin\left(\varphi_{0}+\varphi_{1}\right)-\sin\varphi_{0}}$$

This distance can be used directly by using the linear measurement read- out and Equations (3) or (5). The *a*-value that the system uses and the proper use of Equation (3) can be checked by switching between the linear measurement mode and the angular measurement mode (even with linear optics).

The deviation that an angular measurement will give due to an unknown offset angle *φ*_0_ depends on the offset and the measured angle. This deviation can be calculated from the difference of Equations (3) and (5). Table 10 gives this deviation for a typical value of *a* = 32.5 mm. The main message is that measuring larger angles using such a system requires some attention.

A certificate may contain the characteristic distance *a* (with uncertainty) as well as the nominal distance *a_n_* the system, the deviations this will give and a check on the proper evaluation of Equation (3). Table 10 may be given as an information/warning to the user. The resolution limits are set by the resolution and short-range linearity of the system. A 5 nm displacement nonlinearity and *a* = 32.5 mm corresponds to an angular non-linearity of 0.15 μrad ≈ 0.03″. 

### 7.3. Calibration of Straightness Optics

**Figure 10:**
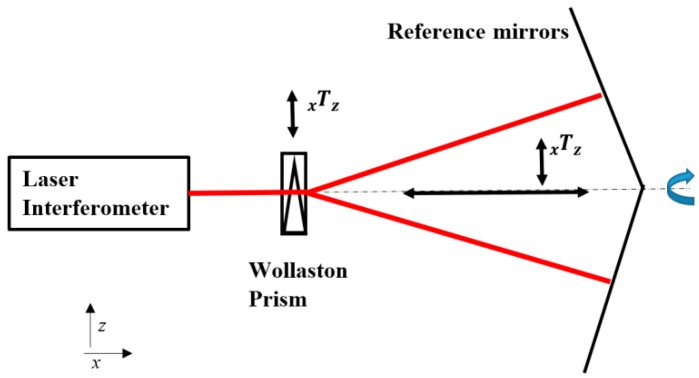
Straightness measurement optics.

The straightness of a slide way may be measured using the so-called ‘straightness optics’. This consists of a Wollaston prism and an assembly of two reflectors, see Figure 10. The properties to be calibrated are the calibration factor and the linearity of the straightness deviation measurement itself, and the absolute straightness of the horizontal line, i.e., ‘what is the straightness of a line that the systems measures to be perfectly straight’. 

For this measurement, it must be mentioned that the sensitivity is far lower than for linear measurements: for 1 μm vertical displacement, the optical path difference is about 0.03 μm. This means that the optical path difference is amplified some 36× to obtain the straightness deviation value. Moreover, the noise, the effect of air turbulence and the interpolation errors are also amplified 36×. The same holds for the mirrors that act as the straightness references: a flatness deviation of 10 nm in one of the mirrors is measured as 0.36 μm measured straightness deviation from the horizontal line. This holds for the ‘short straightness’ optics that has a range of about 3 m. There is also a ‘long straightness’ optics with a range of 30 m and a correspondingly further decrease of sensitivity.

The deviation from straightness can be calibrated using a linear displacement device that displaces the Wollaston prism in the z- direction while it is at a stationary position. When calibrating a machine tool, this can be accomplished on-machine by calibrating the vertical displacement at a fixed x-position against the machine tool z-axis reading. For the straightness reference, the flatness of the reference mirrors can be measured, e.g., using a Fizeau interferometer. An alternative is a classical reversal method: the straightness of a slide way is measured and this measurement is repeated with the reference reversed, i.e., rotated 180° around the x-axis as indicated in Figure 10. The mean value of the straightness deviations is the absolute straightness of the guidance and the difference between the measurements is divided by two is the effective straightness deviation of the optics. This method is mentioned by Evans in an overview of reversal methods [45] and was recently applied in a detailed analysis of these optics [46].

### 7.4. Calibration of Squareness Optics

The squareness optics enabled the calibration of the squareness of two slide ways. In addition to the straightness optics, a pentagon prism, also called the ‘optical square’, was used, reflecting the light at 90°, regardless of the direction of the incoming beam. The calibration of a pentagon prism is a classical calibration item that is well treated in the literature [44,47] and is not repeated here.

## 8. Reporting and Discussion

The results for a typical system can be summarized, and can be presented in a report or certificate, as indicated in Table 11. The numerical values are just for illustration.

In Table 11, the sensor calibration results are averaged over their range and the variation inside their range is incorporated in the uncertainty; these ranges should be reported as well. It is assumed that the differences measured on the comparisons bench are well captured in the frequency differences (from nominal) and alignment errors of the bench. Therefore, a deviation ‘0’ is assumed and the measured deviations are included in the uncertainty. Basically, all errors are systematic, except for the random detector noise that is captured in the uncertainty of the counting system, and the random variation in the laser frequency that is incorporated in the uncertainty of the laser wavelength. In the use of the instrument, random variations in the read-out and in environmental conditions must be taken into account.

Normally, the user would like to use the system ‘as is’ and not make corrections. In that case, the correction should be treated as a single standard uncertainty and quadratically added to the uncertainty. In the example case of Table 11 the conclusion could be that this system can be used without corrections assuming a standard uncertainty of 10 nm + 1.6 × 10^−7^ × *l* + 0.02 × *α_m_* × *l*, where *l* is the measured displacement and *α_m_* is the material linear expansion coefficient. 

A similar approach was taken by Esala [48]; however, this is just one of the many feasible possibilities. For example, in ASME B89.1.8-2011 [24], the comparison to a reference system as shortly described in Section 5.4 is highlighted, where the wavelength calibration is omitted as this is assumed to have been done on the reference system already. This can be justified as described earlier. Probably for this reason, this standard is entitled “performance evaluation” rather than calibration, although most of the described procedures were in line with the definition of calibration. Throughout the text, it has been made clear that there are many possibilities and possible reasoning to carry out some calibrations or not, or in some way or another. It can be argued that some of the proposed tests, e.g., the counting system, are enough to be performed just once in the lifetime of a system. On the other hand, the testing of the counting system on a test bench can be considered as an indirect frequency comparison with the reference laser, as proposed in Section 2.2, making the frequency calibration redundant. The refractive index of air calculation may be considered valid for a software version, and can be performed by any user using the NIST online reference software [41]. A stability and noise test, as briefly mentioned in Section 4, does not require special skills or conditions and can be carried out well by the user. It is generally agreed that certain calibrations are needed periodically, namely those of the material temperature, air temperature, air pressure and laser frequency, more or less in this sequence of relevance. It may appear contradictory that for an instrument that establishes the basic traceability in many dimensional measurement laboratories, the frequency/wavelength calibration is considered not to be the major issue—after an initial calibration verifying its proper operation—however, considering the state of technology, this can be considered as the current situation.

## 9. Conclusions

For displacement laser interferometer systems, many good practices have been established and many accredited laboratories deliver adequate services. Nevertheless, well-argued choices can be made regarding the necessity and feasibility of some calibrations. Users can carry out some of the tests themselves, such as the noise and the software checks on environmental conditions, and based on general knowledge and experiences over some decades some calibrations, e.g., the counting system, the optics and maybe even the laser frequency may be carried out just once or at long intervals. However, for the elements of the measuring system for which the supplier guarantees the performance only with periodic re-calibrations, omitting these could effectively break the conditions under which the traceability is established. This commonly applies especially to the environmental sensors.

## Figures and Tables

**Table 1:** Value and uncertainty of the f-dip of an iodine-stabilized laser system in the course of time.

Year	Mean Frequency	Standard Uncertainty	Relative Standard Uncertainty	Iodine Cell Temperature	Modulation Frequency	Reference
	*f_f_* in MHz	*u*/kHz	*u*_rel_	*T*(*I*)/°C	Mod/MHz	
1983	473,612,353.692	161	3.4 × 10^−10^	15.0 ± 1.0	6.0 ± 1.0	[3]
1992	473,612,353.597	12	2.5 × 10^−11^	15.0 ± 0.2	6.0 ± 0.3	[10]
2001	473,612,353.604	10 50	2.1 × 10^−11^ 1.0 × 10^−10^	15.0 ± 0.2 15.0 ± 1.0	6.0 ± 0.3 6.0 ± 1.0	[8]

**Table 2:** Possible ways of reporting a frequency calibration as part of a laser interferometer system calibration.

	Influencing Factor	Deviation	Deviation in Indication of Length *l* Uncertainty Based on *k* = 2
	Wavelength *λ* nominal vacuum wavelength *λ_nom_* = 632.991370 nm	+(7 ± 6) × 10^−9^ × λ_nom_	−(7 ± 6) × 10^−9^ *× l*
or	Frequency *f* nominal frequency *f*_nom_ = 473.612236 MHz	−(7 ± 6) × 10^−9^ *× f*	−(7 ± 6) × 10^−9^ *× l*
or	Wavelength *λ* nominal vacuum wavelength *λ_nom_* = 632.991370 nm	(0 ± 2.4) × 10^−8^ × λ_nom_	(0 ± 2.4) × 10^−8^ *× l*

**Table 3:** Nominal wavelength of several laser types, as used in laser interferometer systems.

Laser Type	Nominal Wavelength in nm	Nominal Frequency *f_nom_* in MHz	Nominal Beat Frequency with Iodine d-dip *f_d_* = 473,612,379.821 ∆*f* = *f_nom_* − *f_d_* in MHz
HP5526 (before 1980)	632.991480	473,612,154	−226
HP5526 (after 1980)	632.991400	473,612,213	−167
HP5528 (before 1990)	632.991393	473,612,219	−161
HP5528 (after 1990)	632.991370	473,612,236	−144
HP5529	632.991354	473,612,248	−132
Renishaw ML10	632.990580	473,612,827	447
Renishaw RLU	632.990000	473,613,261	881
Heidenhain ILM1101	632.991257	473,612,320	−60
SIOS	632.991400	473,612,213	−167
ZYGO	632.991193	473,612,368	−12

Note that ‘HP’ (Hewlett Packard) has been renamed to Agilent and later Keysight.

**Table 4:** Several alternatives to report the result of a software check.

	Influencing Factor	Deviation	Deviation in Indication of Length *l*
	Calculation of *n_a_*	−(8 ± 2) × 10^−8^	+(8 ± 2) × 10^−8^ × *l*
or	Compensation factor *c_a_*	+(8 ± 2) × 10^−8^	+(8 ± 2) × 10^−8^ × *l*
or	VOL factor (HP/Agilent)	+(8 ± 2) × 10^−5^	+(8 ± 2) × 10^−8^ × *l*

**Table 5:** Example of software check of environmental conditions using displacement comparison.

	Tested Laser Interferometer/mm	Reference Laser Interferometer/mm	Drift/mm
Laser readings without compensation	6988.25646	6988.25626	0
Tested laser with compensation Reference without compensation	6986.36065	6988.25578	−0.00048

**Table 6:** Example of a calibration result of a material temperature sensor and the effect on a measured length.

Influencing Factor	Deviation	Deviation in Indication of Length *l*
Material temperature *T_m_*	+(0.07 ± 0.02) °C	+(0.07 ± 0.02) × *α_m_* × *l*

**Table 7:** Example of a calibration result of an air temperature sensor and the effect on a measured length.

Influencing Factor	Deviation	Deviation in Indication of Length *l*
Air temperature *T_a_*	−(0.10 ± 0.10) °C	−(1.0 ± 1.0) × 10 ^−7^ × *l*

**Table 8:** Example of a calibration result of an air pressure sensor and the effect on a measured length.

Influencing Factor	Deviation	Deviation in Indication of Length *l*
Air pressure *p*	−(1.2 ± 0.3) hPa	+(3.2 ± 0.8) × 10^−7^ × *l*

**Table 9:** Example of a calibration result of an air pressure sensor and the effect on a measured length.

Influencing Factor	Deviation	Deviation in Indication of Length *l*
Air humidity *H*	+(20 ± 10) %Rh	+(1.7 ± 0.8) × 10^−7^ × *l*

**Table 10:** Maximum deviation in a measured angle due to zeroing the reading at an angle *φ*_0_ instead of *φ*_0_ = 0.

Measured Angle	Angle When Zeroing *φ*_0_			
	6′	30′	1°	2°
	maximum deviation			
6′	<0.01″	0.02″	0.06″	0.2″
1°	0.06″	0.4″	1.1″	3.2″
2°	0.2″	1.4″	3.2″	8.8″
5°	1.4″	7.5″	17″	39″
10°	5.6″	29″	1′	1′48″
15°	12.8″	1′06″	2′16″	4′05″

**Table 11:** Typical summary table of the result of a laser interferometer calibration.

	Influencing Factor	Deviation and Standard Uncertainty	Deviation in Length Indication *l* and Standard Uncertainty
1	Vacuum wavelength	+(0.07 ± 0.06) × 10^−7^ × λ	−(0.07 ± 0.06) × 10^−7^ × *l*
2	Counting system	<(0.01 µm + 2 × 10^−8^ × *l*)	(0.00 ± 0.01) µm ± 0.2 ×10^−7^ × *l*
3	Air pressure	+(0.4 ± 0.1) hPa	− (1.0 ± 0.3) ×10^−7^ × *l*
4	Air temperature	−(0.06 ± 0.10) °C	−(0.6 ± 1.0) ×10^−7^ × *l*
5	Air humidity	+(20 ± 10) %Rh	+(1.7 ± 0.8) ×10^−7^ × *l*
6	Calculation of air refractive index *n_a_*	−(0.8 ± 0.3) × 10^−7^ × *n_a_*	+(0.8 ± 0.3) × 10^−7^ × *l*
Total deviation in length measurement without material-temperature correction			(0.00 ± 0.01) µm − (0.8 ± 1.4) × 10^−7^ × *l*
7	Material temperature sensor	−(0.02 ± 0.01) °C	−(0.02 ± 0.01) × *α* × *l*

## Supplementary Materials

Datasets of Figure 3 and Figure 7 are available online at https://www.mdpi.com/1424-8220/19/19/4100/s1.
